# Ribosomal Dysregulation in Metastatic Laryngeal Squamous Cell Carcinoma: Proteomic Insights and CX-5461’s Therapeutic Promise

**DOI:** 10.3390/toxics12050363

**Published:** 2024-05-13

**Authors:** Miao Gao, Ting Liu, Kairui Hu, Songling Chen, Shixin Wang, Di Gan, Zhihan Li, Xiaohuang Lin

**Affiliations:** Key Laboratory of Ministry of Education for Gastrointestinal Cancer, School of Basic Medical Sciences, Fujian Medical University, Fuzhou 350108, China; miaomiao055@163.com (M.G.); liuting8026@fjmu.edu.cn (T.L.); hkr59625@fjmu.edu.cn (K.H.); chensongling@fjmu.edu.cn (S.C.); wangshixin@stu.fjmu.edu.cn (S.W.); gandi@stu.fjmu.edu.cn (D.G.); lizhihan@stu.fjmu.edu.cn (Z.L.)

**Keywords:** LSCC, Lymph node metastasis, Proteomics, Ribosome, CX-5461

## Abstract

One of the main barriers to the successful treatment of laryngeal squamous cell carcinoma (LSCC) is postoperative progression, primarily due to tumor cell metastasis. To systematically investigate the molecular characteristics and potential mechanisms underlying the metastasis in laryngeal cancer, we carried out a TMT-based proteomic analysis of both cancerous and adjacent non-cancerous tissues from 10 LSCC patients with lymph node metastasis (LNM) and 10 without. A total of 5545 proteins were quantified across all samples. We identified 57 proteins that were downregulated in LSCC with LNM, which were enriched in cell adhesion pathways, and 69 upregulated proteins predominantly enriched in protein production pathways. Importantly, our data revealed a strong correlation between increased ribosomal activity and the presence of LNM, as 18 ribosomal subunit proteins were found to be upregulated, with RPS10 and RPL24 being the most significantly overexpressed. The potential of ribosomal proteins, including RPS10 and RPL24, as biomarkers for LSCC with LNM was confirmed in external validation samples (six with LNM and six without LNM) using Western blotting and immunohistochemistry. Furthermore, we have confirmed that the RNA polymerase I inhibitor CX-5461, which impedes ribosome biogenesis in LSCC, also decreases the expression of RPS10, RPL24, and RPS26. In vitro experiments have revealed that CX-5461 moderately reduces cell viability, while it significantly inhibits the invasion and migration of LSCC cells. It can enhance the expression of the epithelial marker CDH1 and suppress the expression of the mesenchymal markers CDH2, VIM, and FN at a dose that does not affect cell viability. Our study broadens the scope of the proteomic data on laryngeal cancer and suggests that ribosome targeting could be a supplementary therapeutic strategy for metastatic LSCC.

## 1. Introduction

Head and neck cancers (HNCs) are ranked as the sixth most common type of cancer worldwide, they encompass a diverse range of tumors classified by their site of origin [1,2]. Laryngeal carcinoma (LC) represents a substantial fraction of HNCs, accounting for one third of all such cases [3]. Laryngeal squamous cell carcinoma (LSCC) is estimated to constitute approximately 85% to 95% of all cases of LC [4,5]. Despite advancements in its treatment, the five-year overall survival rate for laryngeal cancer hovers around 50%, primarily due to the occurrence of distant metastases and the development of therapy-resistant local and regional recurrences [6,7]. It is widely acknowledged that the presence of metastasis at the time of diagnosis is a critical prognostic factor for patients with LSCC [7,8].

The initial spread of head and neck squamous cell carcinoma (HNSCC) typically targets the neck lymph nodes [9,10]. Most patients with LSCC present with advanced stages of the disease, and over 60 percent are predisposed to lymph node metastasis (LNM), which is a crucial factor in their prognosis [11]. The presence, number, distribution, and extranodal extension of lymph node metastases are all critical in assessing the risk of distant disease and patient survival. An ipsilateral single-node metastasis can reduce survival rates by half, and contralateral or bilateral involvement can decrease survival by an additional 50% [12,13]. Consequently, preventing and inhibiting lymph node metastasis should be integral to strategies aimed at controlling tumor progression.

Lymph node metastasis is a multi-step process that encompasses invasion, lymphangiogenesis, the spread of cancer cells through lymphatic channels, their transit into the lymph nodes, and their subsequent settlement and proliferation [14]. Over the last few decades, a range of biomarkers implicated in the metastatic cascade of HNSCC have been identified [15]. Key among these are matrix metalloproteinases (MMPs), which are critical enzymes that degrade and remodel the extracellular matrix (ECM), thus facilitating tumor invasion and metastasis [16,17]. CD44, functioning as a cell surface receptor, not only binds to but also potentiates the activity of MMPs [18]. Furthermore, proangiogenic factors, including VEGF and IL-8, along with chemokine receptor-7 (CCR7), have been implicated in the promotion of metastatic disease [19]. Recent findings also suggest a correlation between elevated levels of miR-23a [20] and EPCAM in laryngeal cancer [13], which is associated with an increased risk of lymph node metastasis and poorer prognostic outcomes. There is still a noticeable gap in in-depth omics-level analyses that could elucidate the molecular mechanisms and identify reliable biomarkers of lymph node metastasis in LSCC.

The metastasis of tumor cells is a complex process involving numerous genes, with increasing evidence suggesting that ribosomal proteins (RPs) play a pivotal role [21]. The proliferation in cancer cells demands accelerated protein production, requiring enhanced ribosome biogenesis. This enhancement can be driven by altered signaling, metabolic shifts, and changes in non-coding RNA, leading to increased RNA Pol I (RNA polymerase I) activity [22]. Studies have revealed that RPs such as RPL15 are key in advancing metastasis, particularly in circulating tumor cells (CTCs), through the amplification of protein translation [23]. Moreover, reducing RPL27A or RPL15 in vitro can hinder the movement of breast cancer cells [24]. La-related protein 6 (LARP6) has also been recognized for its role in epithelial–mesenchymal transition (EMT) through affecting the localization of ribosomal proteins (RPs), thereby aiding cell migration [25]. In addition to cytoplasmic ribosomal proteins, the abnormal expression of mitochondrial ribosomal proteins may also promote the invasion and metastasis of tumors [26]. For example, MRPS16 promotes the migration and invasion of glioma cells by activating the PI3K/Akt/Snail axis [27]. The protein levels of MRPL15, MRPL13, and MRPL54 are associated with the recurrence, distant metastasis, and prognosis of breast cancer [28]. Additionally, MRPL38 is more abundant in most metastatic cancer cell lines [29]. Such studies underscore the critical role of ribosomal protein variations in modulating the transition of cancer cells to a state of enhanced migration and invasion.

In this study, we conducted a tandem mass tag (TMT)-based whole-cell proteomic analysis of LSCC to obtain a comprehensive view of the proteins that are dysregulated and their association with lymph node metastasis. A proteome-based bioinformatics analysis highlighted the importance of ribosomal proteins in LSCC’s metastasis to the lymph nodes. Moreover, we confirmed the efficacy of CX-5461, a selective and orally bioavailable RNA polymerase I inhibitor, in inhibiting LSCC cell invasion in vitro.

## 2. Methods

### 2.1. Clinical Tissue Sample Collection

Laryngeal squamous cell carcinoma tissues, along with adjacent normal tissues, were obtained from patients diagnosed with laryngeal cancer who underwent surgical resection at the First Affiliated Hospital of Fujian Medical University. Patients with a history of chemotherapy or radiotherapy were excluded from the study. After gross examination, pathologists selectively excised non-necrotic sections from the resected tumor specimens. Adjacent normal tissues were harvested at least 2 cm away from the tumor margin. Tumor tissues and adjacent normal tissues from the same patients were stored in liquid nitrogen and were also subjected to pathological confirmation via hematoxylin-eosin (HE) staining by pathologists. For the proteomic analysis, subsets of LSCC patients with LNM (*n* = 10) and without LNM (*n* = 10) were selected based on comparable clinical and histopathological characteristics, as well as similar age and gender distributions. The validation cohort included an additional six LSCC patients with LNM and six LSCC patients without LNM. The clinicopathological features of the 32 patients are summarized in Table 1.

The ethical committee of Fujian Medical University granted approval for this study, and informed consent was acquired from each participant for the use of their data in this research.

### 2.2. Protein Extraction, Digestion, and Tandem Mass Tag (TMT) Isobaric Labeling

Twenty milligrams of tissue were homogenized in lysis buffer [8 M urea, 150 mM NaCl, 50 mM Tris-HCl (pH 8.0), and 1× protease inhibitor (Thermo Fisher Scientific, Waltham, MA, USA, 78429)] and incubated for 30 min on ice. The samples then underwent sonication to reduce lysate viscosity. After centrifugation at 16,000× *g* for 10 min, the soluble supernatants were collected. The protein concentration in the lysates was quantified using BCA assays.

For tryptic digestion, 500 μg of the protein samples was first reduced with 10 mM dithi-othreitol (DTT) at 37 °C for 60 min and alkylated with 50 mM iodoacetamide (IAA), in the dark, for 10 min. The proteins were precipitated with a 5-fold volume of ice-cold acetone, and the resulting pellet was collected via centrifugation at 1000× *g* for 10 min, followed by three washes with precooled acetone. The pellet was then redissolved in 100 mM triethylammonium bicarbonate (TEAB) and digested using trypsin at a 1:50 (*w*:*w*) trypsin-to-protein ratio and incubated at 37 °C overnight.

The peptide concentrations were determined using BCA assays. Subsequently, 100 μg of peptides from each sample were labeled with TMTpro-18plex (A52047) or TMT-11plex (A37725) isobaric tags, according to the manufacturer’s protocol. The 40 samples, comprising 20 tumor-to-normal pairs, included mixed peptides that served as an internal reference across three TMT experiments (the samples’ labeling details are presented in Table S1). The labeled peptides were then mixed, desalted, and vacuum-dried.

### 2.3. Fractionation of Peptides and LC-MS/MS Analysis

Using a high pH reversed-phase peptide fractionation kit (Thermo Fisher Scientific, Waltham, MA, USA, 84868), the labeled peptides were divided into 15 fractions and dried via vacuum centrifugation. Each peptide fraction was dissolved in 0.1% formic acid and injected into an Acclaim PepMap C18 column (75 μm × 25 cm) for LC-MS/MS analysis. A 50-min gradient was run at 200 nL/min on an EASY-nLC 1200 UPLC system (Thermo Fisher Scientific, Waltham, MA, USA); 2% to 30% of solvent B (0.1% formic acid in 98% acetonitrile) was increased to 50% within 5 min and then to 80% over a further minute, where it remained for 4 min. The peptides underwent ionization using an NSI source before a tandem MS analysis using an Orbitrap Exploris 480 MS, coupled online with the UPLC. The settings included a 2.3 kV spray voltage, a funnel RF level of 50, and a capillary temperature of 320 °C. In its data-dependent acquisition (DDA) mode, the full MS operated with a resolution of 60,000 at *m/z* 200, an AGC target of 300%, and scanned a mass range of 350–1600 *m/z*. The fragment spectra were established using a 200% AGC target, 15,000 resolution, and 50 ms injection times, using a Top12 approach. Additional parameters included an intensity threshold of 2 × 10^5^, an isolation width of 1.6 *m/z*, and a normalized collision energy of 30%.

### 2.4. MS Data Processing and Data Analysis

The mass spectrometric files were processed using MaxQuant (version 2.4.2.0, Max-Planck Institute of Biochemistry, Munich, Germany). The data were searched against the Homo sapiens Uniprot database (20,597 sequences), assuming the digestion enzyme trypsin. The mass error was set to 10 ppm for precursor ions and 4.5 ppm for fragment ions. The oxidation of methionine and the protein’s N-terminus acetylation were specified as variable modifications. For the TMT-labeled experiment, the carbamidomethyl of cysteine, TMT of lysine, and the N-terminus were specified as fixed modifications, while, for the TMTpro-labeled experiments, the fixed modifications were set as TMTpro lysine and the N-terminus and carbamidomethyl of cysteine. The number of max missed cleavage sites was set to 2. The enzyme was set as trypsin. The acceptance criterion for identifications was that the false discovery rate (FDR) should be less than 1% for peptides and proteins. For the quantification of proteins, the MS intensity of each protein across three TMT experiments was corrected with respect to the reference channel. The harmonized data from three TMT experiments were subsequently combined into a single expression matrix, subjected to a log2 transformation, and normalized using upper quartile normalization.

### 2.5. Bioinformatics Analysis

The filtered proteomic data with no missing values (n = 5545 proteins) were used as input data for the differential expression analysis. Proteins with a fold change > 1.2 and *p* < 0.01, as determined by the two-sample Student’s *t*-test, were identified as differentially expressed proteins (DEPs). A functional enrichment analysis, including gene ontology (GO) and the Kyoto Encyclopedia of Genes and Genomes (KEGG), was performed using DAVID bioinformatics resources [30]. A Benjamini–Hochberg-adjusted *p*-value of less than 0.05 was considered to indicate statistical significance. A gene set enrichment analysis (GSEA) was carried out to pinpoint the predominantly enriched pathways using the “clusterProfiler” package in R software (version 4.3.2) [8], with pathways achieving a nominal q-value below 0.01 being deemed statistically significant. The Kaplan–Meier plotter, utilizing the R package “survival”, was employed to compare the OS and PFS times between specific groups. The processed gene expression profiles and clinical attributes of laryngeal cancer patients were acquired from the TCGA and CPTAC databases through LinkedOmics (http://www.linkedomics.org, accessed on 10 December 2023). Any samples deficient in crucial clinicopathological or prognostic data were eliminated from the subsequent analyses.

### 2.6. Western Blotting

The proteins from LSCC tissues were isolated as previously described (Method 2.2). For the cell lines, we harvested and thrice washed cells with PBS. They were then lysed on ice for 30 min in RIPA buffer supplemented with phenylmethylsulfonyl fluoride (PMSF) and protease and phosphatase inhibitors. Afterwards, protein separation was conducted via SDS-PAGE, and their transfer onto a PVDF membrane followed. The mem-brane underwent blocking with 5% nonfat milk before overnight incubation with primary antibodies at 4 °C. The employed primary antibodies included GAPDH (Abclonal, Wuhan, China, AC001), β-actin (Abclonal, A17910), RPS10 (Abclonal, A6056), CDH1 (Abclonal, A24874), CDH2 (Abclonal, A19083), Snail (Abclonal, A5243), RPL29 (Immunoway, Plano, TX, USA, YN0379), RPS28 (Immunoway, YN4647), RPL3 (Immunoway, YT4105), RPS26 (Proteintech, Wuhan, China, 14909), VIM (Proteintech, 10366), FN (Proteintech, 15613), ZO1(Proteintech, 21773), and RPL24 (Proteintech, 17082). After being rinsed with PBS and 0.1% Tween 20, the membrane was incubated with the pertinent secondary antibodies for an hour at room temperature. Protein bands were visualized using enhanced chemiluminescence (ECL) reagents and the Bio-Rad ChemiDoc MP imaging system (Hercules, CA, USA), with their quantification performed using ImageJ software (version 1.49).

### 2.7. RNA Isolation and Quantitative Real-Time PCR

Total RNA was isolated from the cells using TRIzol reagent and cDNA was synthesized from 1 μg of total RNA using a reverse transcription kit (YEASEN, Shanghai, China) according to the manufacturer’s instructions. A quantitative mRNA analysis was performed using SYBR Green qPCR Mix (Vazyme, Najing, China). The mean Ct for each sample was normalized using GAPDH as the reference gene (for primer sequences, see Supplementary Materials, Table S2).

### 2.8. Immunohistochemistry

Tissue samples were fixed in 10% neutral buffered formalin for 24 h and then embedded in paraffin. Sections with a thickness of 5 μm were deparaffinized and rehydrated, with their endogenous peroxidase activity quenched using a 3% hydrogen peroxide solution. Sections were stained with anti-PRS10 or RPL24 antibodies at a 1:200 dilution ratio and incubated overnight at 4 °C. After washing with PBST, they were treated with the Elivision super HRP (Rabbit) IHC Kit (Maxim, Fuzhou, China) according to the manufacturer’s protocol. For the visualization of their proteins, diaminobenzidine (DAB kit, Maxim) was applied, and the slides were counterstained with hematoxylin. Imaging was carried out using a Nikon light microscope at 200× magnification. A semiquantitative analysis of protein expression was conducted using Image Pro Plus software (version 6.0).

### 2.9. Cell Lines and Culture

The Tu686 and Tu212 LSCC cell lines were purchased from the Cell Center of Life Science of the Chinese Academy of Science (Shanghai, China). The cells were maintained in RPMI-1640 medium (Gibco, Carlsbad, CA, USA) supplemented with 10% fetal bovine serum and 1% penicillin–streptomycin (100 μg/mL) at 37 °C in a humidified incubator (Thermo Fisher Scientific, Waltham, MA, USA) in the presence of 5% CO_2_.

### 2.10. Cell Viability and Invasion

Cells were seeded onto 96-well plates and left overnight, before being treated with a dose–response-determined volume of CX-5461 (MEC, Lansdale, New Jersey, USA, 13323A) for a duration of 96 h. Cell viability was assessed using a Cell Counting Kit-8 (CCK8) (YEASEN, Shanghai, China, 40203ES). For the invasion assay, cells were placed in serum-free RPMI 1640 media within Matrigelcoated chambers (8 μm pore size, Corning Inc., New York, NY, USA), and the lower chamber was filled with 600 µL of medium supplemented with 20% FBS. After a 48 h incubation at 37 °C with 5% CO_2_, the cells were fixed with 4% paraformaldehyde (PFA) and stained with 0.5% crystal violet for 20 min. The invasive cells were imaged and quantified across five randomly selected fields.

### 2.11. Wound Healing Assay

A wound healing assay was employed for assessing cell migration. Cells were placed in a 6-well plate and left to grow overnight until they covered 80–90% of the surface. A single layer of cells was then scratched using a sterile 10 μL pipette tip and cleaned with PBS to remove any cell residue. After the scratch was made, cells were allowed to grow further in RPMI-1640 culture medium without fetal bovine serum. The movement of cells was observed and captured every 24 h using an inverted microscope.

### 2.12. Statistical Analysis

In the proteomic analysis, we applied a two-tailed unpaired Student’s *t*-test to determine the statistical significance of the difference between LSCC tumor tissues with and without lymph node metastasis (LNM). For comparisons between tumor samples and their adjacent normal tissue counterparts, a two-tailed paired Student’s *t*-test was applied. Proteins with a fold change > 1.2 and a *p* value < 0.01 were defined as significantly differentially expressed. In vitro experiments, such as the transwell assays, wound healing assays, cell viability measurements, and Western blot analyses of various markers, were each independently repeated at least three times. Two-tailed Student’s *t*-tests were used to compare between groups. Data were presented as means ± SD, and *p* < 0.05 was considered significant.

## 3. Results

### 3.1. Schematic Workflow for Screening Metastasis-Specific Proteins from LSCC Patients

**Figure 1:**
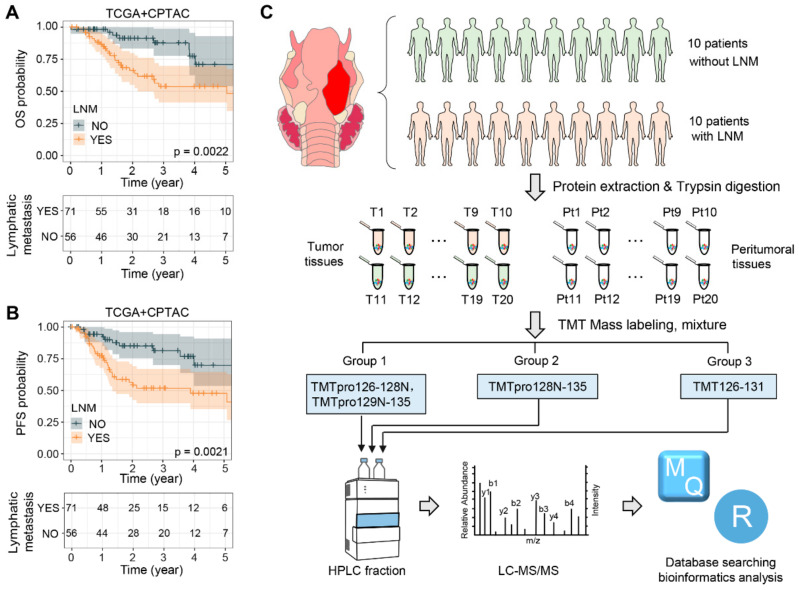
Study design and data analysis pipeline. (**A**,**B**) Kaplan–Meier curves illustrating the overall survival (OS) (**A**) and progression-free survival (PFS) (**B**) of patients in the CPTAC and TCGA LSCC cohorts, stratified by the presence or absence of lymphatic metastasis. (**C**) Workflow and strategy for the quantitative proteomic analysis of LSCC. Abbreviations: LNM, lymph node metastasis; T, tumor tissue; Pt, peritumoral tissue; TMT, tandem mass tag; HPRP, high-performance liquid chromatography; MS, mass spectrometry.

The survival rate of laryngeal squamous cell carcinoma (LSCC) patients is significantly affected by the tumor’s high invasiveness and its tendency to spread to distant body sites, such as cervical lymph nodes and other organs. In our analysis of the clinical data from LSCC patients within the TCGA and CPTAC databases, we discovered that more than 59% of these patients experience lymph node metastasis (LNM). Those with lymph node metastasis showed lower overall survival (OS) and progression-free survival (PFS) rates, as indicated by the generated Kaplan–Meier curves, which had *p* values of 2.2 × 10^−3^ and 2.1 × 10^−3^, respectively (Figure 1A,B, Supplementary Materials, Table S3).

To gain a comprehensive understanding of LSCC patients with lymph node metastasis (LNM), we developed an efficient workflow to identify the proteins that are dysregulated specifically in relation to the LNM occurring in LSCC (Figure 1C). Paired tumor and adjacent non-tumor tissue samples from twenty LSCC patients, including ten with LNM (Patients 1–10) and ten without LNM (Patients 11–20), were collected for a global proteomic analysis utilizing a TMT-based strategy that followed stringent criteria. The clinicopathological characteristics of these 20 LSCC patients are summarized in Table 1. After protein extraction, trypsin digestion, and TMT labeling, the labeled peptides were pooled for HPLC fractionation and subsequently analyzed using LC-MS/MS. In order to avoid protein variations due to batch effects, the patients were randomly shuffled during sample preparation and MS acquisition, and the abundance of each protein was normalized, with respect to a reference channel, across three TMT groups (Groups I–III). The database search results indicated that the labeling efficiency of all three TMT groups exceeded 98% (Figure S1A) and that the overall intensities of each channel fluctuated within a small range (Figure S1B). These findings demonstrate the rigorous control we had over our experimental procedure and attest to the high quality of the proteomic data.

### 3.2. The Landscape of Dysregulated Proteins in LSCC Patients with LNM

**Figure 2:**
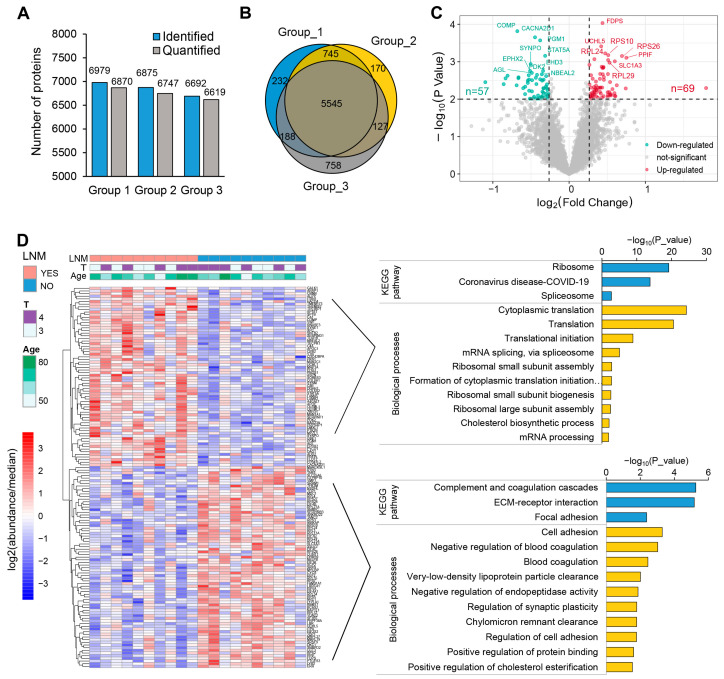
Quantitative profiling of whole-cell proteome in LSCC. (**A**) Summary of identified and quantified proteins in the three TMT groups. (**B**) Venn diagram of the quantified proteins of three TMT-based protein profiling: 5545 proteins were quantified across all samples. (**C**) Volcano plot indicating the differentially regulated proteins in LSCC with LNM versus LSCC without LNM. Red and blue colors represent fold changes ≥ 1.2 and a *p* < 0.01. (**D**) Heatmap showing the differently expressed proteins. Histogram showing the enrichment of KEGG and GOBP (GO biological processes) terms in upregulated (**upper right**) and downregulated (**bottom right**) proteins; this analysis was conducted using DAVID. Detailed data are available in Tables S4 and S5.

For the quantitative proteomics analysis, three sets of TMT experiments identified a total of 6979, 6875, and 6692 proteins, respectively, with 6870, 6747, and 6619 of these proteins being quantified (Figure 2A). Among these proteins, 5545 were quantified across all samples and, therefore, included in subsequent analyses (Figure 2B, Table S4). Next, we compared the differentially expressed proteins between tumors with and without LNM, revealing that 126 proteins were significantly differentially expressed (fold change > 1.2, *p* < 0.01): 69 were upregulated and 57 downregulated, as shown in Figure 2C. The 69 stably upregulated proteins in tumors with LNM could serve as a potential pool for the identification of high-invasion-specific LSCC biomarkers. Furthermore, 23% of these proteins (16 out of 69) exhibited the same trend of dysregulation when comparing tumor to peritumoral tissues (Figure S2B). To better understand the biological functions of the dysregulated proteins specific to LNM, we performed Gene Ontology (GO) and Kyoto Encyclopedia of Genes and Genomes pathway (KEGG) enrichment analyses using DAVID bioinformatics resources. The most enriched pathways among the proteins positively related to LNM included ribosome (*p* = 6.31 × 10^−20^), spliceosome (*p* = 1.96 × 10^−3^), and cytoplasmic translation (*p* = 5.53 × 10^−25^), indicating an extraordinary activation of ribosome biogenesis and the synthesis of proteins. As expected, the proteins negatively related to LNM were significantly enriched in pathways such as complement and coagulation cascades (*p* = 5.54 × 10^−6^), ECM–receptor interactions (*p* = 6.76 × 10^−6^), and cell adhesion (*p* = 5.16 × 10^−4^) (Figure 2D, Table S5). This enrichment indicates a disruption of the intercellular matrix and cell–cell junctions, which, coupled with the active biosynthesis of tumor cells, collectively promotes the invasion and metastasis of LSCC.

The dysregulated proteins in T/Pt are displayed as a volcano plot (Figure S2A); a total of 848 proteins were observed to be significantly differentially expressed (a fold change > 1.2, *p* < 0.01), of which 419 were upregulated and 429 were downregulated. The most enriched pathways of the dysregulated proteins in tumor tissues are illustrated in Figure S2C. Notably, upregulated proteins were overrepresented in pathways including DNA replication (*p* = 1.35 × 10^−4^), spliceosome (*p* = 1.18 × 10^−3^), base excision repair (*p* = 2.95 × 10^−3^), and antigen processing and presentation (*p* = 8.16 × 10^−3^), while the downregulated proteins were enriched in pathways including complement and coagulation cascades (*p* = 4.54 × 10^−12^), thermogenesis (*p* = 2.65 × 10^−7^), and oxidative phosphorylation (*p* = 3.23 × 10^−7^). This enrichment analysis underscores the notable genomic instability and mitochondrial functional impairments in LSCC cells.

### 3.3. Dysregulation of Ribosomal Proteins is Closely Associated with LNM of LSCC

**Figure 3:**
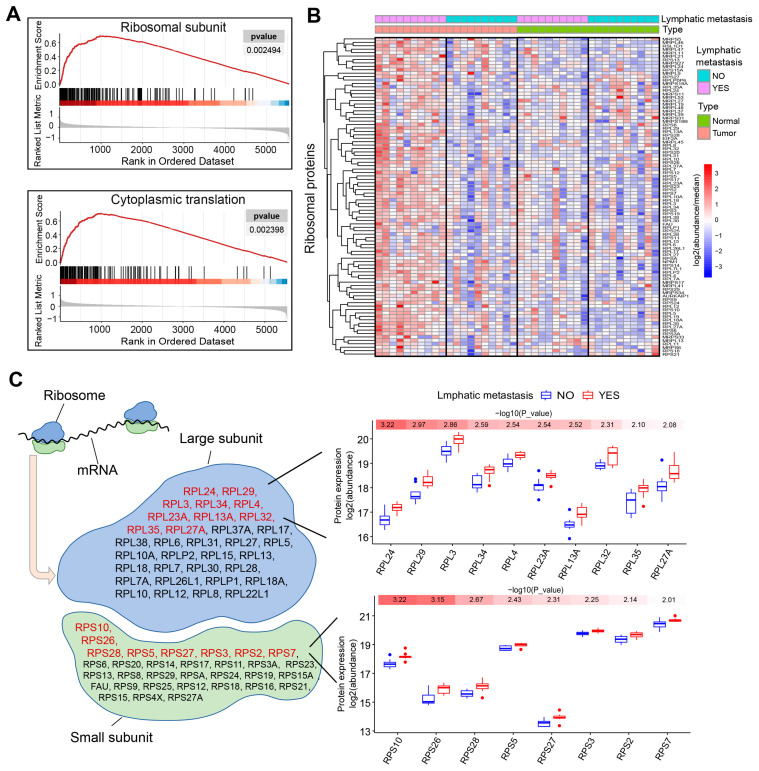
The ribosome is markedly activated in LSCC with LNM. (**A**) Gene set enrichment analysis (GSEA) of samples with and without LNM. (**B**) Heatmap showing the expression pattern of ribosomal proteins in LSCC with and without LNM. (**C**) The quantified ribosomal proteins have been categorized according to their presence in either the large or small subunit. Proteins specifically associated with lymph node metastasis and exhibiting a *p*-value of less than 0.01 are marked in red. To the right, box plots illustrate the expression levels of these proteins, arranged in ascending order according to their *p* values.

The aberrant growth and proliferation of tumor cells depend on increased protein synthesis, which requires an overly activated ribosomal biogenesis process. In line with this requirement, the most enriched pathways among the dysregulated proteins in LSCC with LNM are all ribosome-associated. Our gene set enrichment analysis (GSEA) reveals that most of the proteins of the ribosomal subunits, as well as those related to translation, are upregulated to various extents in LSCC with lymph node metastasis (Figure 3A). When all quantified ribosomal proteins were displayed as a heatmap, the ribosomal protein expression levels in LSCC tissues with lymph node metastasis were generally found to be higher than those in adjacent non-cancerous tissues (Figure 3B). In contrast, the fluctuations in ribosomal protein expression between cancerous and peritumoral tissues in LSCC without lymph node metastasis were found to be less pronounced.

The ribosome consists of 60S and 40S subunits, which include a series of proteins and RNA molecules. These subunits assemble to form a specific structure that accommodates mRNA and tRNA and promotes reactions such as amino acid binding. Ten proteins, comprising up to 21% of the proteins quantified in the 60S ribosomal subunit, and eight proteins, representing 25% of those in the 40S subunit, were significantly overexpressed in LSCC with lymph node metastasis, exhibiting a *p*-value of less than 0.01, as shown in Figure 3C. These 18 stably upregulated proteins could serve as a potential resource for the identification of LSCC biomarkers specific to lymph node metastasis. Notably, RPS10 and RPL24, which are integral to the small and large ribosomal subunits, respectively, are the proteins most relevant to lymph node metastasis.

### 3.4. Validation of the LNM Specific Biomarkers

**Figure 4:**
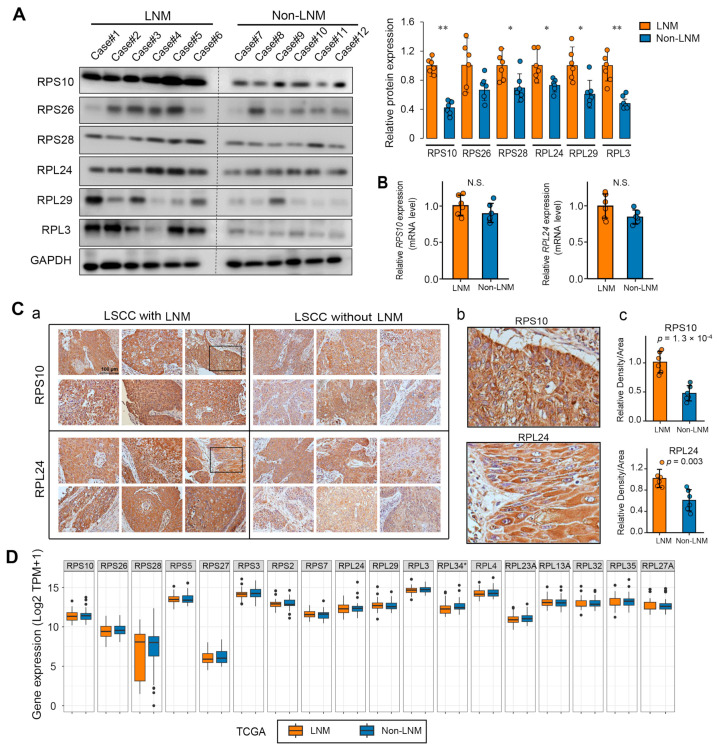
Validation of biomarker expression patterns in LSCC with LNM. (**A**) Western blot validation of the protein abundance of the most upregulated ribosomal proteins in LSCC with LNM (*n* = 6) compared to LSCC without LNM (*n* = 6). Densitometric quantification of Western blot signals is illustrated in the histogram on the right. (**B**) QPCR analysis detected the expression of *rps10* and *rpl24* genes in LSCC with (*n* = 6) versus without LNM (*n* = 6). (**C**) Representative IHC staining demonstrated marked overexpression of RPS10 and RPL24 in LSCC with LNM: (**a**) Original magnification, ×20; (**b**) representative higher magnification (×40) images of the views marked by the black squares in (**a**), showing the hybridization of RPS10 and RPL24 to tumor cells; (**c**) assessment of proteins expression by relative density to area. (**D**) Gene expression of ribosomal proteins specifically associated with LNM. The mRNA expression data from LSCC cases with (*n* = 53) and without (*n* = 39) LNM were extracted from the TCGA database and compared. Abbreviations: LNM, LSCC with lymph node metastasis; NO, LSCC without lymph node metastasis; TPM, transcripts per million. Data are presented as mean ± SD. Significant differences were determined using a two-tailed *t*-test, * *p* < 0.05, ** *p* < 0.01.

To validate the biomarkers identified via MS, we procured an additional 12 LSCC tissue samples, thereby extending our study beyond the initial cohort of 20 patients. Within this new subset, half the subjects exhibited lymph node metastasis, while the other half were metastasis-free. We focused on the ribosomal proteins previously identified as the most upregulated (RPS10, RPL24, RPS26, RPL29, RPL3, and RPS28). A Western blot analysis confirmed that these proteins had heightened expression specificity in LNM within this novel validation cohort (Figure 4A). To address any potential bias in protein quantification due to variations in tumor purity, we conducted immunohistochemical (IHC) analyses for RPS10 and RPL24, which were consistent with our Western blot results (Figure 4C).

To determine whether the heightened expression of these ribosomal proteins was rooted in gene expression anomalies, we performed quantitative PCR (QPCR) assays for the *rps10* and *rpl24* genes and found no significant alterations (Figure 4B). This finding implies that the overexpression of ribosomal proteins in LSCC is likely an event that occurs during translation or post-translation, rather than at the gene transcription stage. In pursuit of further evidence, we analyzed the expression of the genes encoding these ribosomal proteins in LSCC cases from The Cancer Genome Atlas (TCGA) database. In agreement with our experimental results, there were no notable disparities at the mRNA level in the identified ribosomal proteins with LNM-specific overexpression (Figure 4D). This additional analysis reinforces the notion that post-transcriptional mechanisms may account for the discrepant ribosomal protein expression observed in LSCC with lymph node metastasis.

### 3.5. CX-5461 Hinders Ribosome Biogenesis and Reduces the Expression of Several Ribosomal Proteins

**Figure 5:**
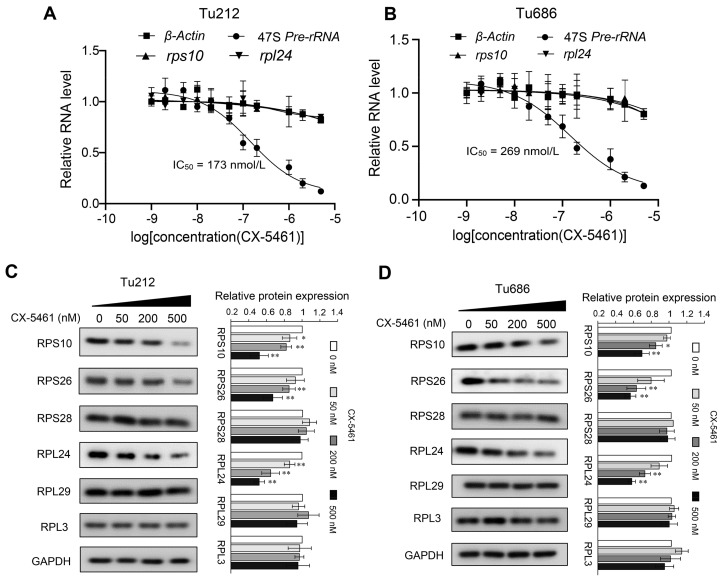
Inhibition of rRNA production and ribosomal protein expression by CX-5461. (**A**,**B**) qRT-PCR analyses of 47S pre-rRNA, β-actin, rps10, and rpl24 transcription after a 2 h treatment of CX-5461 in Tu686 (**A**) and Tu212 cells (**B**); (**C**,**D**) Western blot detecting the expression of six ribosomal proteins RPS10, PRS26, RPS28, RPL24, RPL29, and RPL3, after a 48 h treatment of CX-5461 in Tu686 (**C**) and Tu212 cells (**D**). Data are presented as mean ± SD, n = 3. Significant differences were determined using a two-tailed *t*-test, * *p* < 0.05, ** *p* < 0.01. Abbreviations: IC50, half-maximal inhibitory concentration.

CX-5461 is an orally available inhibitor of ribosome biogenesis capable of disrupting the production of rRNA and thereby inhibiting ribosome biogenesis within the nucleolus. Based on our previous findings of increased ribosomal activity in LSCC tissues with lymph node metastasis compared to those without metastasis, we proposed that CX-5461 could potentially inhibit the abnormal activation of ribosomes in laryngeal cancer, thereby impeding cancer cell invasion and metastatic progression. To test this hypothesis, we conducted quantitative PCR experiments to assess the effectiveness of CX-5461 in inhibiting rRNA synthesis in laryngeal cancer cells. The results showed that CX-5461 significantly reduces the production of 47S rRNA precursor in vitro at nanomolar concentrations, with a mean half-maximal inhibitory concentration (IC50) of 173 nM in the Tu686 cell line and 269 nM in the Tu212 cell line. The primary target of CX-5461 is RNA polymerase I, which is involved in rRNA transcription, rather than RNA polymerase II, which is responsible for mRNA transcription [31]. Consequently, CX-5461 does not suppress the mRNA expression levels of the genes encoding RPS10 and RPL24, as shown in Figure 5A,B.

To investigate the effects of CX-5461 on the expression of RPs at the protein level, we conducted Western blot analyses on laryngeal cancer cell lines treated with varying con-centrations of CX-5461. The results indicated that CX-5461 significantly reduced the expression levels of RPS10, RPS26, and RPL24, while not affecting RPS28, RPL29, and RPL3 (Figure 5C). The differing sensitivities of various ribosomal proteins to CX-5461 suggest that the dynamic regulation of ribosomal proteins in cells is complex and may involve multiple layers of regulatory mechanisms. Given the strong correlation of RPS10, RPL24, and RPS26 with LNM in LSCC, and as their expression is inhibited by CX-5461, we suggest that CX-5461 has the potential to be used to treat metastatic LSCC.

### 3.6. CX-5461 More Effectively Inhibits LSCC Invasion than cell Viability

**Figure 6:**
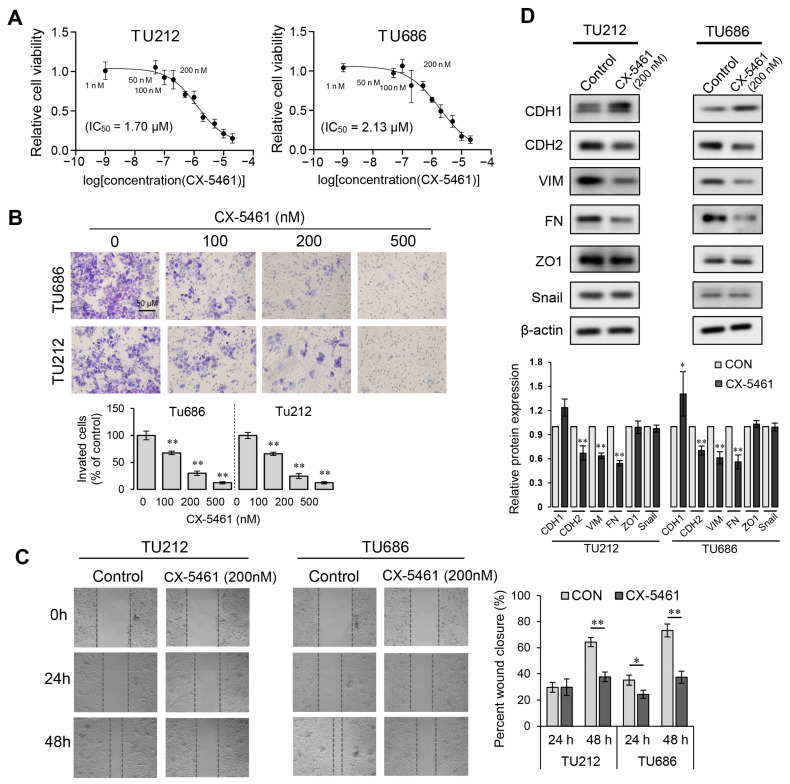
CX-5461’s inhibition of cell viability and invasion. (**A**) Effect of CX-5461 on the cell viability of Tu686 and Tu212 cells. (**B**) Transwell invasion assays were conducted on Tu686 and Tu212 cells using various concentrations of CX-5461, as indicated. (**C**) Wound healing assay revealing the inhibition of cell migration by CX-5461. (**D**) Western blot analysis detecting the effect of CX-5461 on the expression levels of EMT markers CDH1, CDH2, VIM, FN, ZO1, and Snail. Data are presented as mean ± SD, n = 3. Significant differences were determined using a two-tailed *t*-test, * *p* < 0.05, ** *p* < 0.01.

Next, we sought to investigate the anti-proliferative and anti-invasive potential of CX-5461 against laryngeal squamous cell carcinoma (LSCC). By performing the CCK-8 assay, we assessed the reduction in the cell viability of the Tu686 and Tu212 cell lines across a range of drug concentrations. Although CX-5461 indeed inhibited the viability of these laryngeal cancer cells, they displayed a relative insensitivity to it when compared to other cell lines, such as HCT116 and A375, which had IC50s in the low nanomolar range (hundreds or even tens of nM) [31]. The IC50 values of CX-5461 were notably higher for Tu686 and Tu212, at 2.13 μM and 1.71 μM, respectively (Figure 6A). Nevertheless, CX-5461 appears to have a significant inhibitory impact on the metastatic potential of laryngeal cancer cells. The results of the transwell assay show that CX-5461 concentrations of 100 nM or higher significantly reduced the invasiveness of both the Tu686 and Tu212 cell lines (Figure 6B). Interestingly, cell viability was not significantly affected by CX-5461 concentrations of 100 nM and 200 nM. Moreover, this effective concentration aligns precisely with the one required to diminish the expression levels of the ribosomal proteins RPS10 and RPL24 (Figure 5C). To further confirm the specific inhibitory ability of CX-5461 towards cell invasion and metastasis, we assessed the effects of a 200 nM CX-5461 treatment on scratch closure and the expression of epithelial–mesenchymal transition (EMT) markers in laryngeal cancer cells. The results indicated that treatment with 200 nM CX-5461 significantly inhibited cell migration (Figure 6C), promoted the expression of the epithelial cell marker E-cadherin (CDH1), and suppressed the expression of the mesenchymal cell markers N-cadherin (CDH2), vimentin (VIM), and fibronectin (FN) (Figure 6D).

## 4. Discussion

In this study, we report a comprehensive quantitative proteomic analysis of laryngeal squamous cell carcinoma, focusing on the proteome dysregulation associated with lymph node metastasis.

The potential of clinical proteomics to identify and quantify new biomarkers, as well as to distinguish patient profiles, offers remarkable promise for early molecular detection, prognostication, and tailored therapeutic strategies. However, the integration of proteomic technologies into routine clinical practice remains limited, despite the proven success of oncological identification through multi-omics research in specific cancer cohorts [32,33,34]. Proteogenomic data releases for common cancers, such as breast [35], lung [36], liver [37], and HNSCC [38], have deepened our understanding of tumor biology. Huang et al. contributed to this by providing an extensive proteogenomic database for HNSCC that included multi-omics data from 49 LSCC cases, focusing on distinguishing cancerous from non-cancerous tissues [38]. However, the mechanisms and biomarkers of laryngeal cancer risk, such as lymph node metastasis, vascular invasion, and tumor grading, have not yet been fully elucidated. To address this gap, our study implemented a proteomic workflow on a precisely matched cohort of 20 LSCC patients, evenly divided between those with and without lymph node metastasis and controlled for gender and age. In contrast to the proteomic data published by Huang et al., in which cancerous and non-cancerous tissues were not paired, our proteomic data include expression profiles from both cancerous and paired adjacent normal tissues. This meticulously designed proteomic analysis, which carefully considers sample selection, not only provides a refined view of metastatic laryngeal cancer but also enriches the breadth of the available data on laryngeal cancer.

Our comparative proteomic analysis identified a set of 848 proteins with marked expression differences between LSCC tissues and their normal counterparts. The enrichment analyses of these proteins highlighted several critical pathways, with DNA replication emerging as the most prominent, along with a significant overexpression of the spliceosome, cell cycle, and ribosome pathways (Figure S2). These insights reflect the tumor’s proliferative vigor and align well with prior proteomic research into HNSCC [38,39]. The consistency of these findings affirms the authenticity of our proteomic data and the appropriateness of our sample selection.

A key finding of our study is the identification of 126 aberrantly expressed proteins in LSCC with lymph node metastasis. Proteins that are downregulated primarily enrich pathways related to cell adhesion (Figure 2D), corresponding to the metastatic tumor’s tendency to detach from the primary site and spread distantly [40]. This pattern of protein function enrichment has also been observed in proteomic analyses of metastatic colorectal cancer [41] and lung cancer [42]. Notably, among the 69 overexpressed proteins in the lymph node metastasis group, 18 belong to ribosomal subunits. The majority of the other quantified ribosomal proteins are also overexpressed, albeit with slightly lower fold-changes than these 18 (Figure 3). Research has shown that ribosomes can modulate the rate of protein synthesis and play critical roles in cellular processes, such as proliferation, differentiation, apoptosis, and transformation [43,44]. Tumors may enhance ribosomal function to support their increased demand for active biosynthesis [45,46]. The overactivation of ribosomes in tumors is regulated by oncoproteins, noncoding RNAs, and various other factors [47]. Myc is one of the proto-oncogenes involved in abnormal ribosome biogenesis by promoting the transcription of rDNA [22,48]. Conversely, compromised ribosome function can inhibit the malignant progression of tumors [49]. Mossmann et al. have suggested that targeting abnormal ribosome biogenesis by blocking mTORC1/ribosomal protein S6 (RPS6) signaling using drugs is an effective cancer treatment strategy [50]. Our proteomic data suggest that this ribosomal overactivation may play a particularly prominent role in driving the metastasis of laryngeal cancer and could be considered a diagnostic marker for metastatic laryngeal carcinoma.

Among the aberrantly expressed ribosomal proteins in LSCC tissues with lymph node metastasis, RPS10 and RPL24 are significantly prominent and are considered biomarkers for lymph node metastasis. Mutations in RPS10 have been reported to correlate with Diamond–Blackfan anemia [51] and to have a role in regulating the mitochondrial function in plants [52]. Additionally, RPL24 may play a role in liver regeneration and could serve as a potential prognostic biomarker for cervical cancer when treated with cisplatin and concurrent chemoradiotherapy [53,54]. Both RPS10 and RPL24 have consistently shown their higher expression in the LNM group in external validation cohorts, as demonstrated by our WB and IHC assays (Figure 4). However, the qPCR and TCGA analyses identified no significant differences in their mRNA levels between the groups with and without LNM. These findings suggest that post-transcriptional regulatory mechanisms, such as translation efficiency or protein control by the ubiquitin proteasome system and autophagy, may play roles in the ribosomal hyperactivation observed in metastatic laryngeal cancer cells [55]. We noted that RPL15, a ribosomal protein well known to be overexpressed and to enhance tumor invasiveness in metastatic breast cancer [23,56], did not exhibit significant changes in our data, indicating its potential ribosomal heterogeneity across different tumor types. Recent research has proposed the concept of ribosomal heterogeneity, implying that ribosomes are not uniform entities, but rather variable complexes with components that differ across diseases and cellular states [57]. Variations can occur in rRNA modifications [58,59], ribosomal protein ratios [60], post-translational alterations [57], and their associated protein regulation, leading to the formation of “specialized ribosomes” or “onco-ribosomes” in cancer. Our findings may support this evolving viewpoint.

Given our discovery of heightened ribosomal activity in metastatic laryngeal cancer cells, we propose that inhibiting ribosome biogenesis may effectively suppress the invasion and metastasis of such cancer cells. We experimentally employed CX-5461, an inhibitor of ribosome biogenesis [31,61], and observed its potent ability to suppress ribosomal RNA transcription in laryngeal cancer cell lines. Intriguingly, it also attenuated the protein expression levels of RPS10, RPL24, and RPS26, irrespective of their mRNA expression. We speculated that this CX-5461 treatment diminishes the expression of essential rRNA, promoting the cellular proteostasis mechanisms, such as the ubiquitin–proteasome system, to degrade unincorporated ribosomal proteins [55,62]. Although CX-5461 has shown significant anti-proliferative effects on various cancer cell lines, including HCT116 and A375 [31], laryngeal cancer cells exhibit a relative resistance to it, with an IC50 value nearly 10 times higher than that of HCT116 cells.

Our in vitro experimental results demonstrate that CX-5461 preferentially inhibited the invasiveness of laryngeal cancer cells, rather than their viability (Figure 6). This suggests that CX-5461 could potentially serve as an adjunct to frontline therapies or as a prophylactic agent to prevent postoperative recurrence, rather than as the primary treatment for killing laryngeal cancer cells. This conclusion requires further validation through more extensive in vivo experiments in the future. Moreover, for CX-5461 to be applied in the treatment of laryngeal cancer, its validation through more extensive preclinical and clinical research is necessary. Considering that CX-5461 has been linked to potential DNA damage [61] and topoisomerase II poisoning [63], it is particularly essential for future studies to conduct comprehensive in vivo toxicological assessments of CX-5461 using animal models. It is noteworthy that, apart from CX-5461, other RNA polymerase I inhibitors such as BMH-22, BMH-21 [64], and POL1-IN-1 may also have promising potential applications. It is crucial to compare the efficiency of different ribosome biogenesis inhibitors at inhibiting cancer cell invasion and metastasis both in vitro and in vivo, as well as their safety profiles. There is a pressing demand for novel, safer, and more efficacious treatments targeting ribosomal biogenesis in tumors with elevated ribosomal activity to open new paths for cancer treatment.

In summary, our study characterized the comprehensive proteome of laryngeal carcinoma with lymph node metastasis and analyzed the molecular mechanisms involved. We proposed and demonstrated the value of ribosomal biogenesis as a potential therapeutic target for metastatic laryngeal cancer. We believe that this study offers valuable insights into the progression of LSCC with lymph node metastasis and facilitates advancements in the development of diagnostics and therapeutics for LSCC patients with lymph node metastasis.

## 5. Conclusions

Using a TMT-based proteomic workflow, we depicted the proteomic landscape of LSCC with lymph node metastasis, thereby enriching the current proteomic database of laryngeal cancer. A significant upregulation of ribosomal proteins was noted in metastatic LSCC, with the ribosomal proteins RPS10 and RPL24 identified as potential biomarkers of the condition. By employing CX-5461 as an inhibitor of ribosomal biogenesis, our preliminary in vitro experiments showcased the potential of targeting ribosomal biogenesis as a therapeutic strategy for metastatic laryngeal squamous cell carcinoma.

## Figures and Tables

**Table 1:** Clinical characteristics of the 32 LSCC patients.

Characteristic	Whole Cohort	Lymph Node Metastasis	
		Present	Absent
Total (cases)	32	16	16
Age [years (mean ± SD)]	64.1 ± 8.1	63.6 ± 7.5	64.6 ± 8.7
Gender [cases (%)]			
Male	32 (100)	16 (50)	16 (50)
Pathologic-T [cases (%)]			
T3	15 (50)	8 (53.3)	7 (46.7)
T4	17 (50)	8 (47.1)	9 (52.9)

Abbreviations: SD, standard deviation.

## Data Availability

The data in this study are available on reasonable request from the corresponding author.

## Supplementary Materials

The following supporting information can be downloaded at: https://www.mdpi.com/article/10.3390/toxics12050363/s1, Table S1: Labeling reagents for each sample; Table S2: List of QPCR primers; Table S3: Clinical data of patients in the TCGA-HNSC Larynx cohort and CPTAC-HNSC Larynx cohort; Table S4: The proteome data generated by this study; Table S5: Pathway enrichment analysis of proteins correlated with lymphatic metastasis. Supplementary Figures: Figure S1: Quality control of TMT-base quantitative proteomics; Figure S2: Comparison of proteins between the tumor tissues and peritumoral tissues; Supplementary images: Uncropped full-length Western blots.
