# Supplementary material for: Ribosomal Dysregulation in Metastatic Laryngeal Squamous Cell Carcinoma: Proteomic Insights and CX-5461’s Therapeutic Promise
Source: Toxics. 2024 May 13;12(5):363. doi: 10.3390/toxics12050363 (PMC11126056; doi:10.3390/toxics12050363)
Supplement: Supplementary file 1 [file toxics-12-00363-s001.zip › Supplementary Figures.pdf]

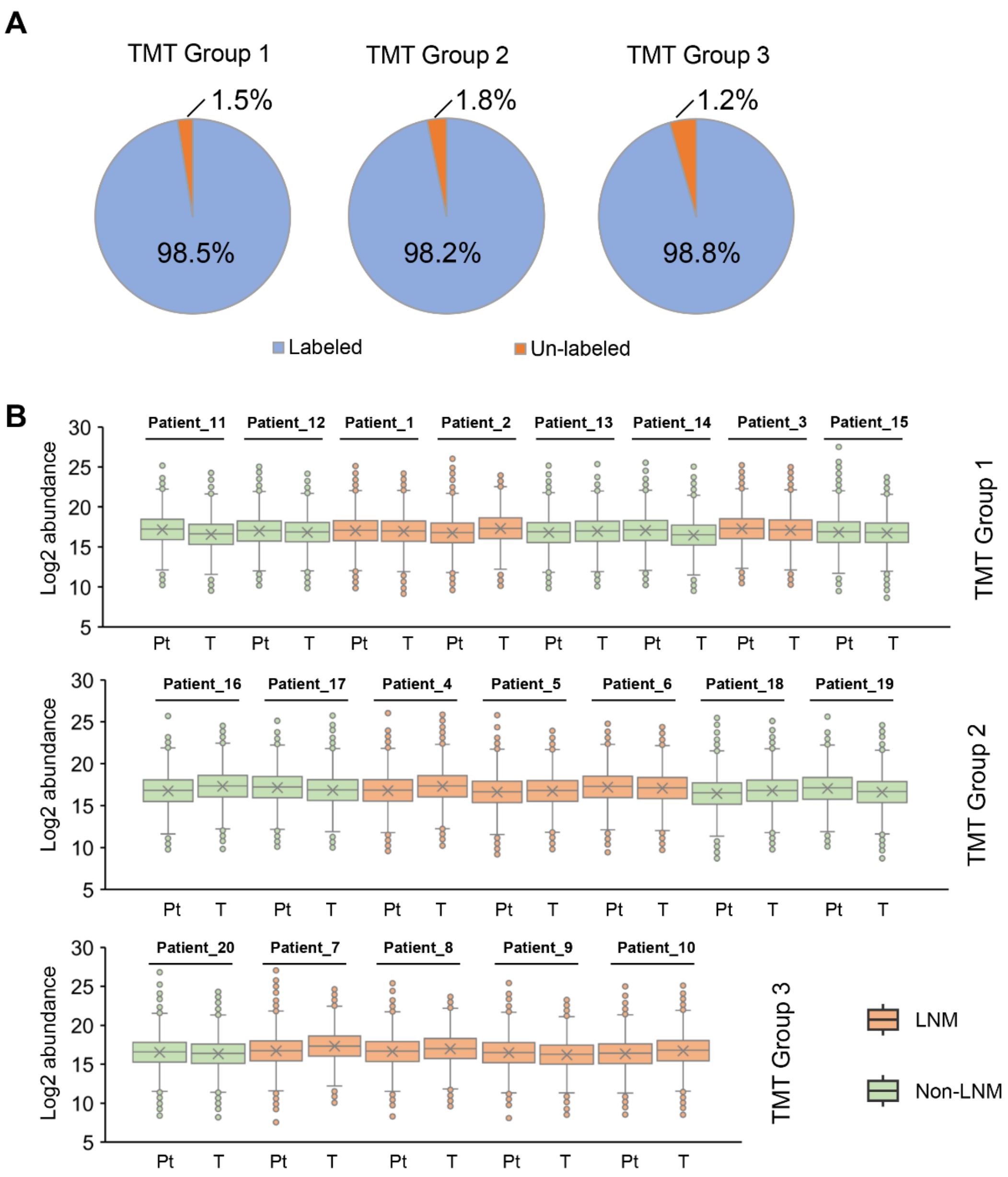

Figure S1. Quality control of TMT-base quantitative proteomics. (A) The pie chart demonstrates the high labeling efficiency of the three TMT experiments. (B) Boxplot displays the distribution of samples in three TMT experiments, as well as the overall intensities for each channels.

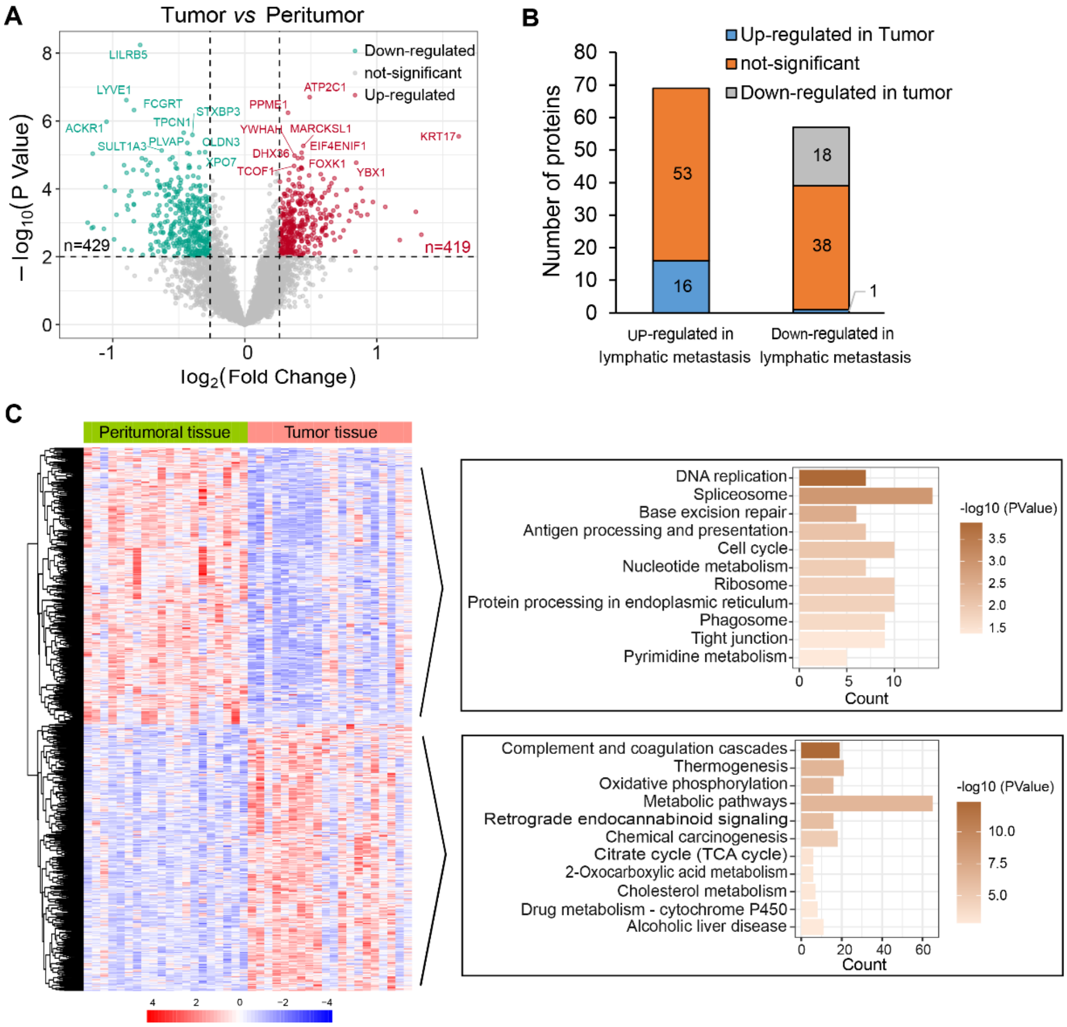

Figure S2. Comparison of proteins between the tumor tissues and peritumoral tissues. (A) Volcano plot indicating proteins with significant abundance differences between tumor and peritumoral tissues. Red and blue colors represent fold change ≥ 1.2 and P < 0.01. (B) An overview of proteomic expression changes in tumor and peritumoral tissues for proteins that are specifically dysregulated in LNM (refer to Figure 2). (C) Heatmap showing the differently expressed proteins. Histogram showing the enrichment of KEGG terms in proteins with upregulated (upper right) and downregulated (bottom right), the analysis was conducted by DAVID service.
