# Supplementary material for: Ribosomal Dysregulation in Metastatic Laryngeal Squamous Cell Carcinoma: Proteomic Insights and CX-5461’s Therapeutic Promise
Source: Toxics. 2024 May 13;12(5):363. doi: 10.3390/toxics12050363 (PMC11126056; doi:10.3390/toxics12050363)

Figure 4A__Oringin images

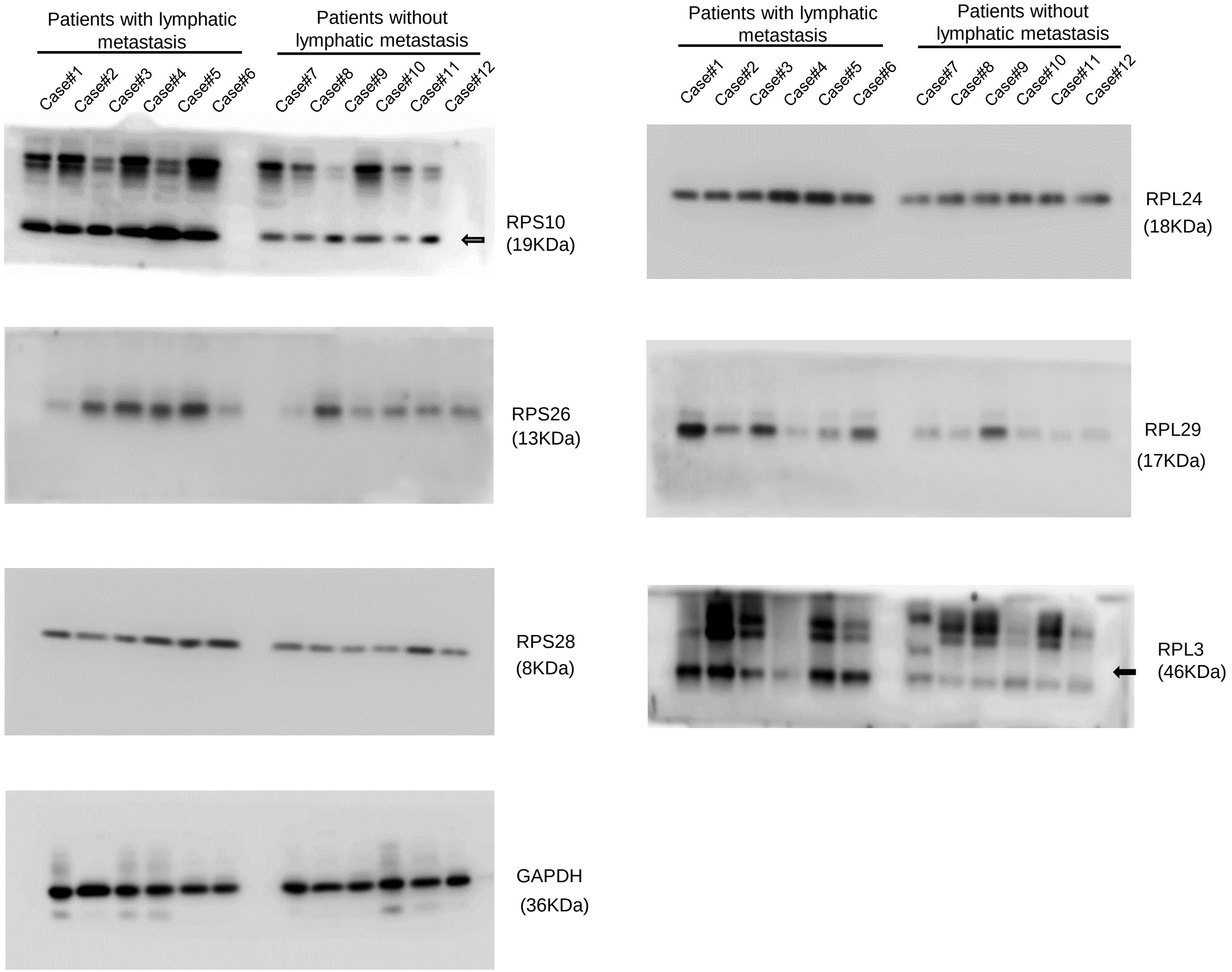

Figure 5C__Oringin images

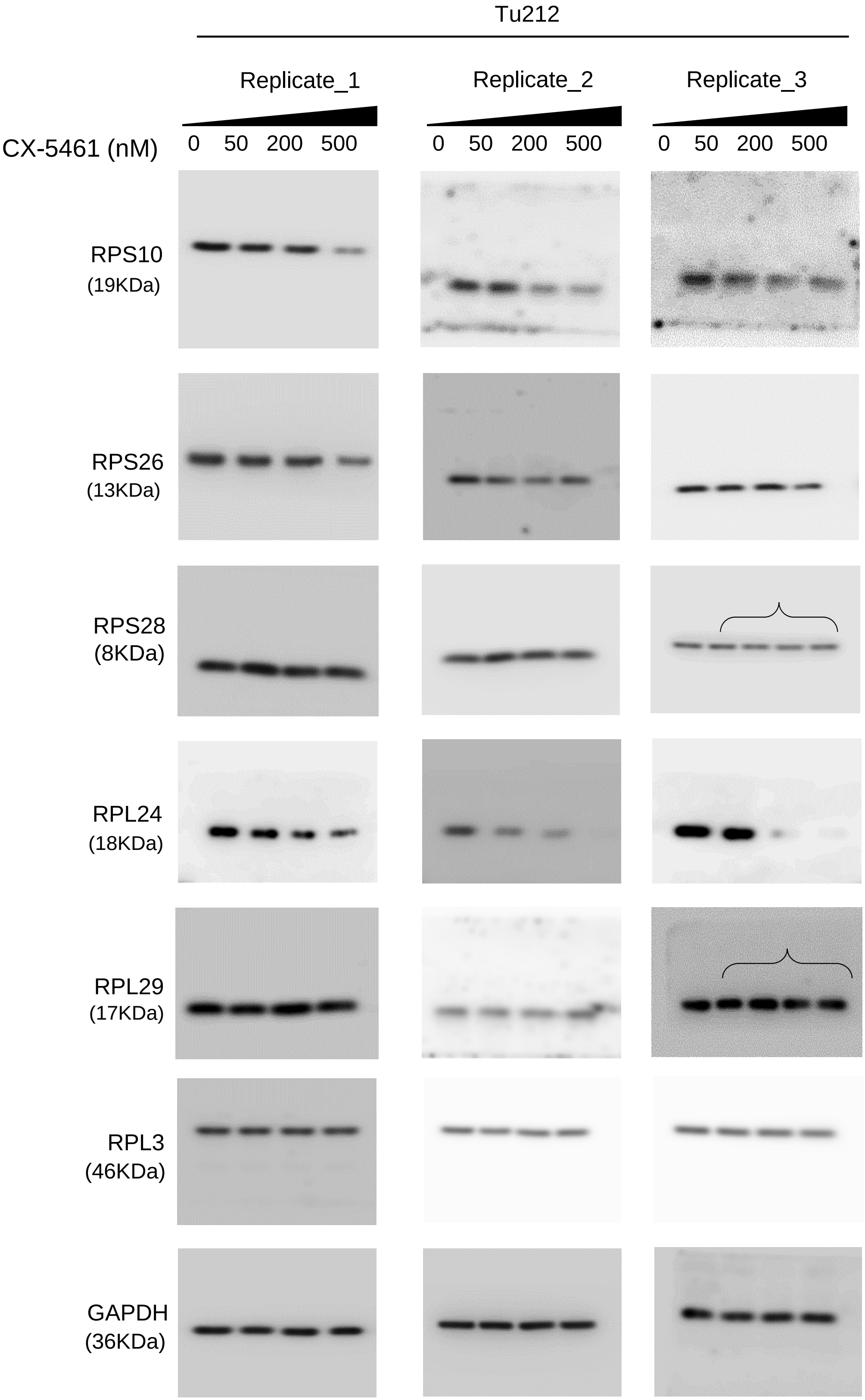

Figure 5D__Oringin images

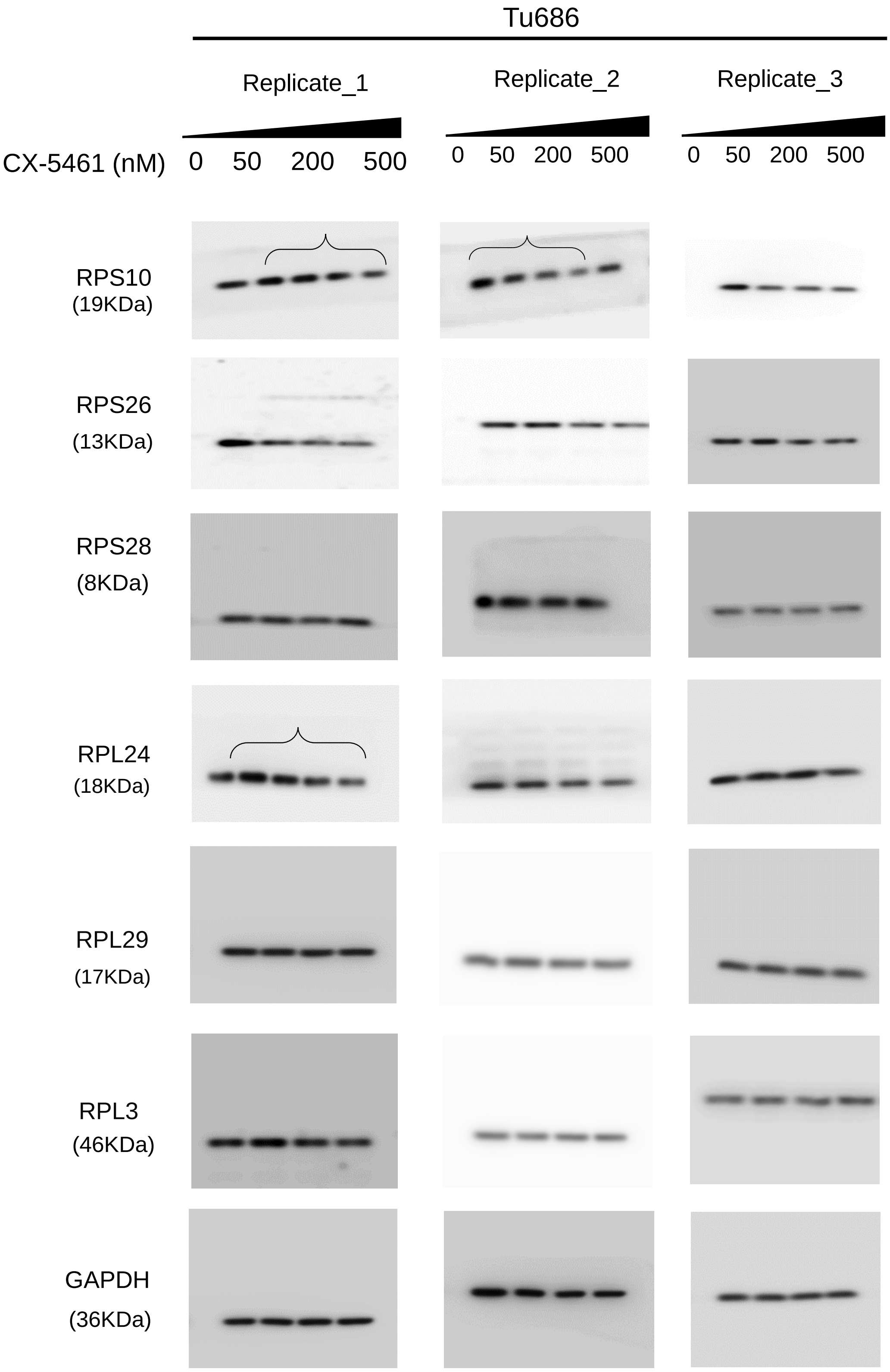

Figure 6D__Oringin images

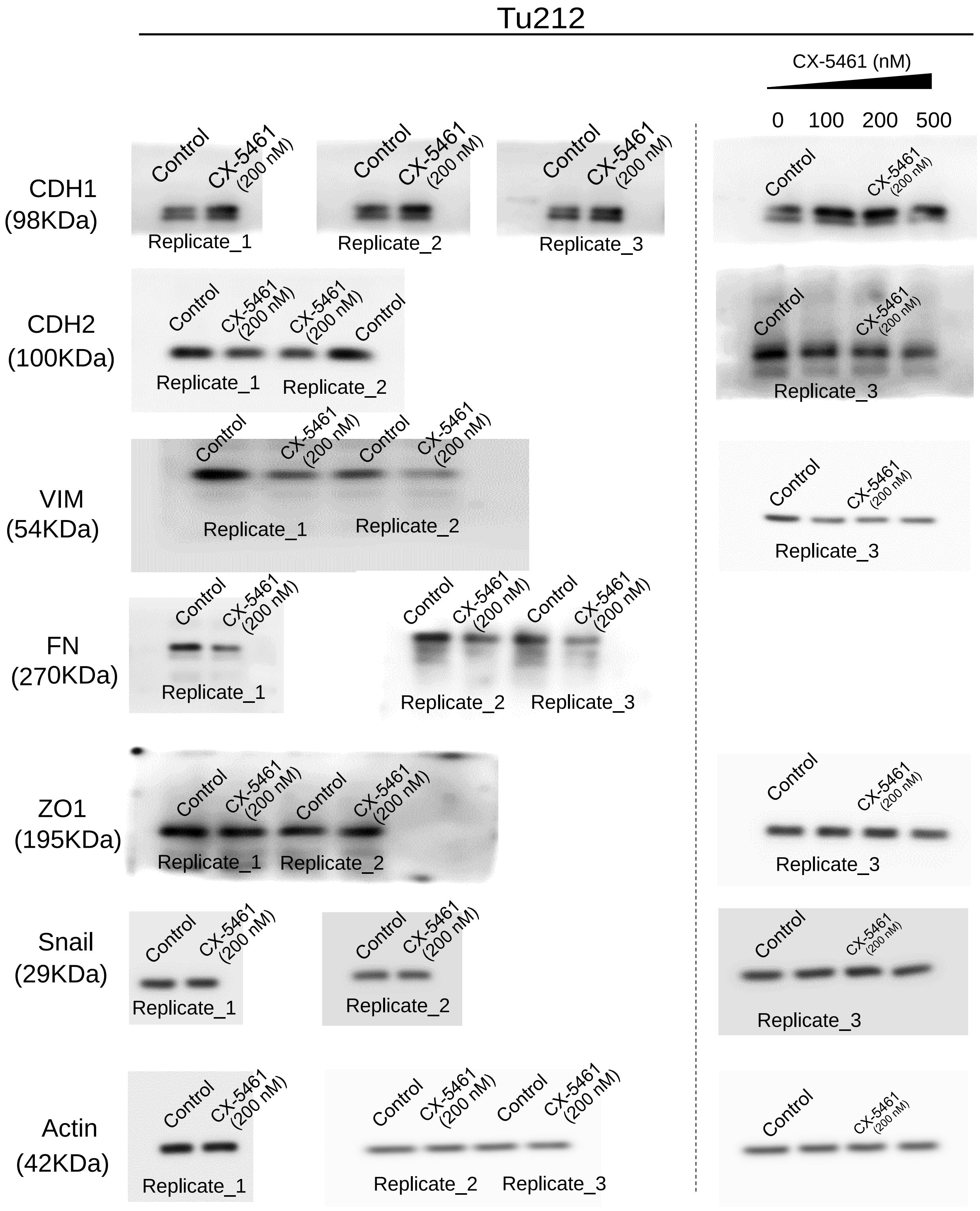

Figure 6D__Oringin images

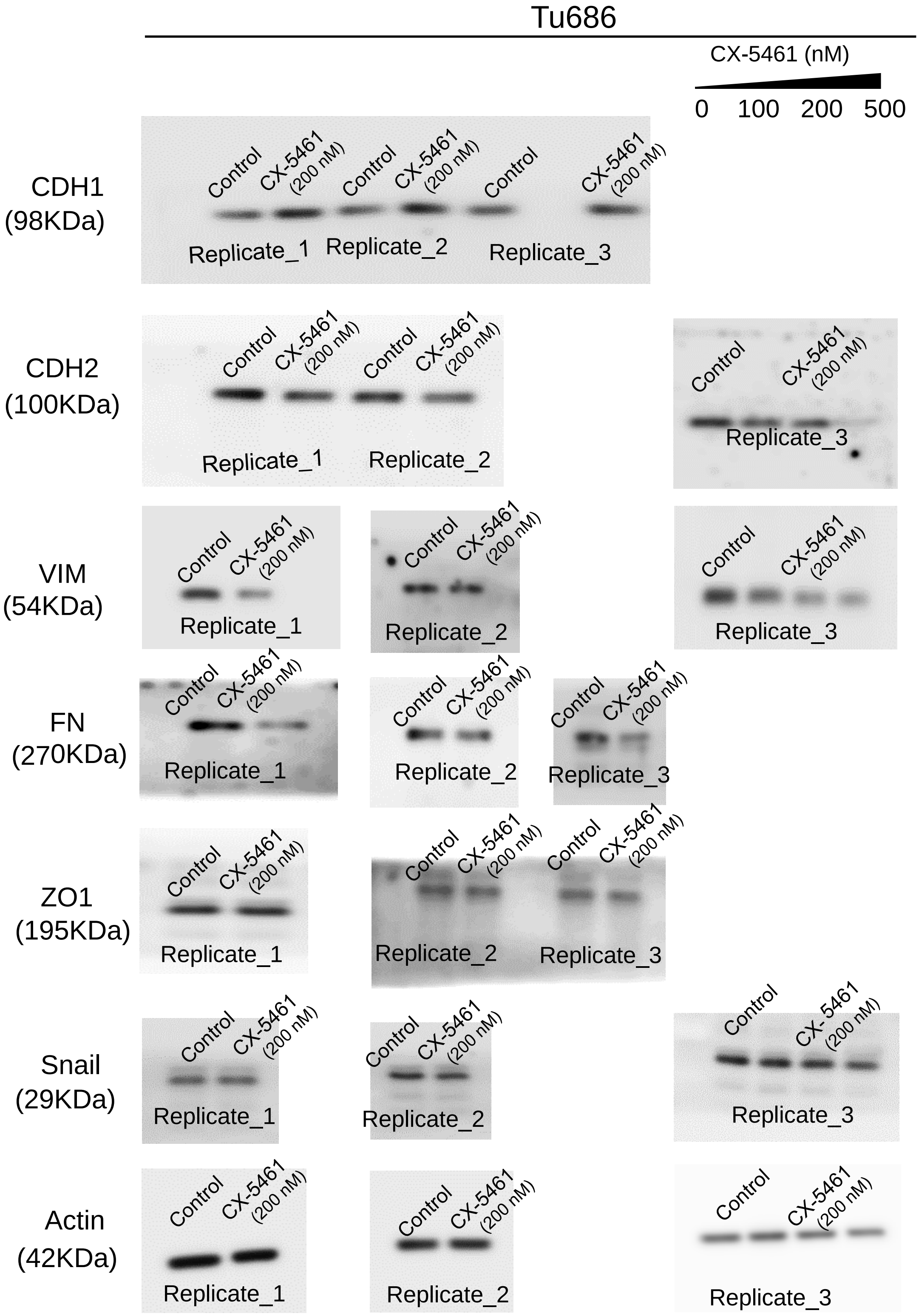

Supplement: Supplementary file 1 [file toxics-12-00363-s001.zip › Supplementary images.pdf]
